# An Emerging Role of TIM3 Expression on T Cells in Chronic Kidney Inflammation

**DOI:** 10.3389/fimmu.2021.798683

**Published:** 2022-01-26

**Authors:** Can Lu, Huihui Chen, Chang Wang, Fei Yang, Jun Li, Hong Liu, Guochun Chen

**Affiliations:** ^1^ Department of Nephrology, The Second Xiangya Hospital, Central South University, Changsha, China; ^2^ Hunan Key Laboratory of Kidney Disease and Blood Purification, Changsha, China; ^3^ Department of Ophthalmology, The Second Xiangya Hospital, Central South University, Changsha, China; ^4^ Clinical Immunology Research Center, Central South University, Changsha, China

**Keywords:** TIM3, T cell, macrophage, infection, chronic kidney inflammation

## Abstract

T cell immunoglobulin domain and mucin domain 3 (TIM3) was initially identified as an inhibitory molecule on IFNγ-producing T cells. Further research discovered the broad expression of TIM3 on different immune cells binding to multiple ligands. Apart from its suppressive effects on the Th1 cells, recent compelling experiments highlighted the indispensable role of TIM3 in the myeloid cell-mediated inflammatory response, supporting that TIM3 exerts pleiotropic effects on both adaptive and innate immune cells in a context-dependent manner. A large number of studies have been conducted on TIM3 biology in the disease settings of infection, cancer, and autoimmunity. However, there is a lack of clinical evidence to closely evaluate the role of T cell-expressing TIM3 in the pathogenesis of chronic kidney disease (CKD). Here, we reported an intriguing case of *Mycobacterium tuberculosis* (Mtb) infection that was characterized by persistent overexpression of TIM3 on circulating T cells and ongoing kidney tubulointerstitial inflammation for a period of 12 months. In this case, multiple histopathological biopsies revealed a massive accumulation of recruited T cells and macrophages in the enlarged kidney and liver. After standard anti-Mtb treatment, repeated renal biopsy identified a dramatic remission of the infiltrated immune cells in the tubulointerstitial compartment. This is the first clinical report to reveal a time-course expression of TIM3 on the T cells, which is pathologically associated with the progression of severe kidney inflammation in a non-autoimmunity setting. Based on this case, we summarize the recent findings on TIM3 biology and propose a novel model of CKD progression due to the aberrant crosstalk among immune cells.

## A Clinical Case Highlights the Involvement of TIM3 in Inflammatory Kidney Injury

**Figure 1:**
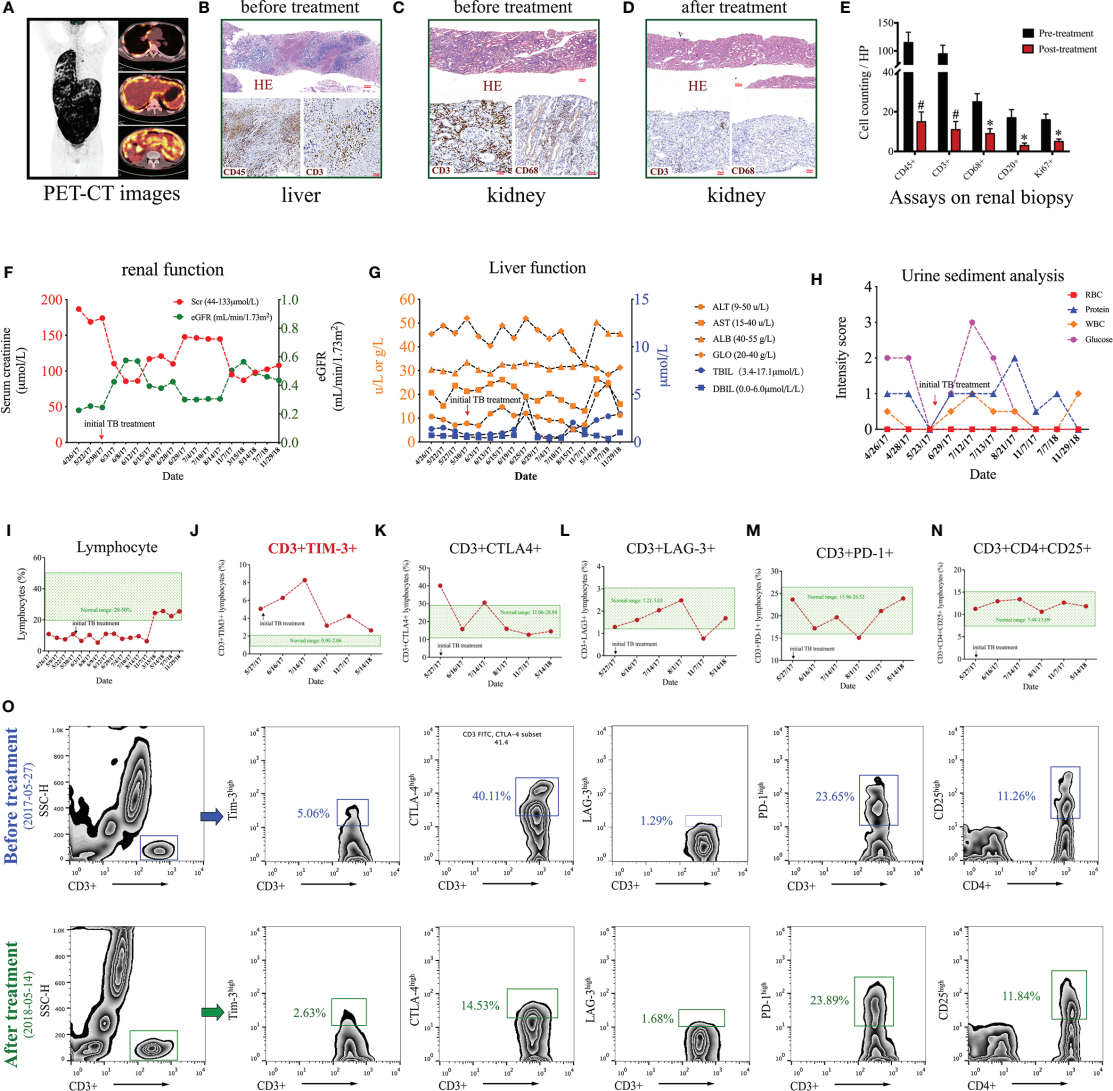
Clinical data of a case of *Mycobacterium tuberculosis* (Mtb) infection. **(A)** PET-CT images. **(B)** Liver biopsy before anti-Mtb treatment with HE and immunohistochemical staining using antibodies against CD45 and CD3. **(C–E)** Renal biopsies before **(C)** and after **(D)** anti-Mtb treatment and quantitative analysis **(E)** on kidney interstitial cells by expressions of CD45, CD3, CD68, CD20, and Ki67. **(F–I)** Time-course of renal function **(F)**, liver function **(G)**, urine sediment **(H)**, and circulating lymphocyte ratio **(I)**. **(J–N)** Time-course of flow cytometry assays on different subsets of circulating T cells, including CD3+TIM 3+ **(J)**, CD3+CTLA-4+ **(K)**, CD3+LAG-3+ **(L)**, and CD3+PD1+ **(M)**, and CD3+CD4+CD25+ **(N)**. **(O)** Gating strategies and representative figures of flow cytometry assays. *P < 0.01, ^#^P < 0.001.

We begin with an intriguing case of *Mycobacterium tuberculosis* (Mtb) infection linking overexpression of T cell immunoglobulin domain and mucin domain 3 (TIM3) on T cells to progressive tubulointerstitial nephritis. A 21-year-old man presented to the renal division with nocturia, fatigue, and weight loss for over one month. On admission, this patient had no typical cough, sputum, and chest pain symptoms. Physical examination revealed tenderness of the upper abdomen and palpable liver and spleen below the costal margin. Blood tests indicated a moderate loss of renal function (Scr 186.9μmol/L). Repeated urinary tests showed significant glycosuria and mild proteinuria. Radiology examinations identified the enlargements of multiple lymph nodes at the retroperitoneum, mediastinum, supraclavicular space, and mesenteric area. PET-CT scan demonstrated extensive hypermetabolic activities in the tissues of the pleura and peritoneum, which were anatomically enriched in the right thoracic cavity and abdomen (**Figure 1A**). This patient suffered from an ongoing systemic inflammatory response and disease progression as indicated by significant elevation of serological C-reactive protein (58.4 mg/L) and erythrocyte sedimentation rate (34 mm/h). Liver biopsy revealed intense infiltration of circulating leukocytes (CD45^+^) and T cells (CD3^+^) in the tissue (**Figure 1B**). Meanwhile, renal biopsy demonstrated minor glomerular abnormalities but typical tubulointerstitial nephritis featuring massive CD3^+^ T cells and CD68^+^ macrophages in the renal interstitial compartment (**Figure 1C**). Bone marrow biopsy showed active proliferation. Comprehensive laboratory screenings for autoimmune, malignancy, and hematological diseases were all negative. No specific findings could be detected by whole-exome sequencing or multiple microbiological cultures. However, the T-SPOT.TB tests were strongly positive in three independent replicates.

This patient was clinically diagnosed with tubulointerstitial nephritis caused by active Mtb infection based on the clinical manifestations/symptoms, the laboratory/radiology results, the biopsy findings, and the exclusion of other common differential diagnoses. According to the WHO guidelines recommended for those with extensive tuberculosis disease (1), this patient received a modified 8-month anti-Mtb regimen using isoniazid, rifampicin, ethambutol, and pyrazinamide. Close clinical monitoring was performed on the liver and kidney function tests after the initiation of therapy. This patient ultimately recovered from the symptoms of tuberculosis infection after the anti-Mtb treatment. The follow-up examinations of both laboratory tests and radiology showed that the abnormal changes in multiple systems were markedly ameliorated or disappeared. A repeated renal biopsy confirmed significant remission of the recruited immune cells, including CD45^+^ myeloid cells, CD3^+^ T cells, and CD68^+^ macrophages (**Figure 1D**). Further quantitative analysis on the renal biopsy revealed that the number of kidney interstitial immune cells, including CD45^+^ leukocytes, CD3^+^ T cells, CD68^+^ macrophages, CD20^+^ B cells, and Ki67^+^ proliferative cells, was all dramatically reduced by anti-Mtb treatment (**Figure 1E**). The effectiveness and safety of anti-Mtb treatment was confirmed by the time-course of blood and urine tests, including renal function (**Figure 1F**), liver function (**Figure 1G**), and urine sediment (**Figure 1H**).

Beyond the abnormal findings described above, we also noted a persistent lymphopenia, from which the patient barely recovered after eight months of anti-Mtb treatment (**Figure 1I**). But the number of leukocytes and neutrophils stayed at normal levels. This highlighted the involvement of lymphocytes in Mtb infection. To further explore the pathogenesis of lymphopenia in this case, time-course analysis of flow cytometry was conducted to characterize multiple subsets of T and B cells in circulation. No significant abnormalities could be detected in the proportion of T helper cells (CD3^+^CD4^+^), cytotoxic T cells (CD3^+^CD8^+^), B cells (CD32^+^CD19^+^), and natural killer cells (CD3^–^CD16^+^CD56^+^). However, further assays of the T cell subsets uncovered that the proportion of CD3^+^TIM3^+^ T cells far exceeded the normal range on admission and peaked (>4-fold) at two months of treatment. It subsequently dropped to a nearly normal range after the end of anti-Mtb treatment (**Figure 1J**). Unexpectedly, several other co-inhibitory/regulatory receptors on T cells, including CD3^+^CTLA4^+^, CD3^+^PD1^+^, CD3^+^LAG3^+^, and CD3^+^CD4^+^CD25^+^, were slightly influenced and mostly remained in the normal ranges during the disease progression (**Figures 1K–N**). The representative figures of flow cytometry before and after the treatment are shown in **Figure 1O**. Of note, the expression patterns of TIM3 on T cells were closely related to the disease development and the prognosis in this case. Collectively, these findings revealed that Mtb infection might induce progressive kidney inflammation remote from the original site of bacterial invasion, which in this case likely occurred in the respiratory or digestive system. To our knowledge, this is the first clinical report to demonstrate time-course expression of TIM3 on T cells, which participate in progressive kidney inflammation in a non-autoimmunity setting.

## The Rationale of Targeting T Cell Dysregulation in Chronic Kidney Inflammation Upon Infection

Regardless of its etiology, chronic kidney disease (CKD) progression is pathologically featured by a cumulative infiltration of circulating immune cells (2–4). Based on current knowledge, it is generally believed that the initial trafficking of circulating immune cells into the involved kidneys of CKD intends to eliminate pathogenic factors, clear out necrotic cells and damaged tissues from the original insults, and expectantly facilitates nephron recovery (5, 6). In this context, transient activation of the recruited immune cells is beneficial because they are helpful to eliminate the pathogenic factors. Ideally, these activated immune cells produce abundant chemokines to establish a pro-inflammatory milieu and meanwhile secrete a series of anti-inflammatory cytokines and growth factors to promote inflammation resolution and tissue repair. However, the well-tuned action of the immune system could be undermined in certain conditions, resulting in development of chronic kidney inflammation and CKD progression.

Although the pathophysiological mechanisms are not fully understood, multiple lines of clinical evidence have revealed infection as one of the major risk factors that exacerbates CKD progression in different kidney disease settings (7–9). In this process, T cells are known to play a central role in regulation of both innate and adaptive immune response against infection. Upon pathogens stimulation, effector T cells exert their inflammatory functions by directly killing infected host cells, activating innate immune cells, and secreting cytokines (like IFNγ) to promote tissue inflammation against invasive microbes. At the same time, activation of regulatory T (Treg) cells dampens the inflammatory response to avoid excessive tissue damage by their robust production of inhibitory cytokines (like IL10) (10, 11). In most cases, invasive pathogens can be successfully cleared out or inactively confined in the involved organ. Therefore, T cells synergistically orchestrate the inflammation against exogenous pathogens and the subsequent restoration of immune homeostasis for tissue repair. Under some conditions of chronic infection, however, prolonged pathogenic stimulation continuously promotes the activation of effector T cells. This ultimately results in a state of T cell dysfunction, which is also termed T cell exhaustion (12–14). T cell dysfunction has been widely described in many experimental and clinical settings of chronic infections, including hepatitis B virus (HBV) (15), hepatitis C virus (HCV) (16), human immunodeficiency virus (HIV) (17, 18), and Mtb (19). Of clinical significance, secondary kidney involvements commonly occurred in most of these infectious diseases (20).

Mechanistically, microorganism can induce kidney damage by direct invasion on the site or deposition of circulating antibody-antigen immune complexes in the condition of chronic infection (21). As presented in this case, however, chronic infection might also trigger progressive kidney inflammatory response by its interference with the immune system, which is undermined by tangly crosstalk between T cells and myeloid immune cells (22, 23). In this context, T cell dysregulation due to prolonged exposure of exogenous pathogens promotes the pro-inflammatory phenotype of multiple effector cells, which migrate into the involved kidneys remote from the original infection site and contribute to persistent inflammatory tissue injury (24, 25). Importantly, the “inflamed” phenotype of peripheral T cells might sustain for a long time and affect the tissue inflammatory homeostasis after they migrate from the circulation into the involved kidney (26). Therefore, physicians need to comprehensively assess the clinical significance of T cell-mediated crosstalk among immune cells and consider the therapeutic potentials of targeting T cell activity in CKD progression with a timing-specific manner.

## TIM3 Expression Differentially Modulates T Cell Response During Infection

As observed in this case, T cell dysfunction in chronic infection is generally characterized by sustained expressions of co-inhibitory receptors, such as CTLA4, PD1, TIM3, LAG3, and TIGIT (12–14). There is a consensus that these co-inhibitory receptors can regulate T cell biology through at least three different ways, including directly inhibiting the effector activation, promoting the regulatory capacities, and modulating the inflammatory response of innate immune cells. In many cases, these co-inhibitory receptors on T cells are critical for maintaining immune homeostasis as they counterbalance co-stimulatory signals and prevent an excessive inflammatory response to pathogen stimulation (27). However, it is important to note that some functional effector T cells also express the inhibitory receptors. Under certain conditions, co-expression of inhibitory receptors on T cells could promote an inflammatory response (14). Several key controversies and questions remain open about the biology of co-inhibitory receptors on T cells in the field of nephrology, such as whether and how the kidney disease setting or renal microenvironment might differentially dictate the expression pattern of these co-inhibitory receptors and their exerting functions upon infection, particularly in a cell-type-specific manner.

As one of the major members of the co-inhibitory receptors, TIM3 was initially identified on the activated Th1 cells as a transmembrane marker that negatively modulated innate and adaptive inflammatory responses (28, 29). Later, emerging studies uncovered that TIM3 was also constitutively expressed on many other cell types, including Treg cell (30), Th17 cell (31), macrophage (32), dendritic cell (33, 34), natural killer (NK) cell (35), and mast cell (36, 37). A large amount of studies have revealed TIM3 as a potent regulator of the immune system that closely correlates to the activities of multiple human diseases, such as infection, cancer, autoimmunity, and transplant tolerance (38–44). This review mainly focuses on TIM3 function in the context of infectious diseases. Of clinical importance, TIM3 expression in the T cell compartment is largely restricted to inflammatory IFNγ-producing effector T cells and FOXP3+ Treg cells, known as major participants in most of human infectious and inflammatory diseases. In most cases of chronic infection, TIM3 serves as an inhibitory receptor with a key role in regulating IFNγ-mediated inflammation. For example, TIM3 overexpression undermines the Th1/Tc1 immunity in disease progression while inhibition/blockade of TIM3 signaling rescues dysfunctional T cells in multiple infection settings, such as HCV (45, 46), HBV (47), HIV (48), and lymphocytic choriomeningitis virus (LCMV) (49). Moreover, TIM3 expression also indirectly exerts its inhibitory functions on the effector Th1/Tc1 cells by significantly enhancing the suppressive capacity of FOXP3+ Treg cells (30, 40, 50, 51).

Apart from its known inhibitory effect on Th1-type immunity, however, our case leaves open the question of whether and how TIM3 overexpression might distinctly contribute to the pathogenesis of chronic kidney inflammation during infection. Indeed, the emerging evidence supports that TIM3 serves as a co-stimulatory receptor rather than a dominant inhibitor in Th1 cell activation under certain circumstances. TIM3 exerts essential functions for optimal T cell immunity by promoting short-lived effector T cells but suppressing memory precursors (52). Interestingly, the absence of TIM3 in CD8+ T cells significantly impaired the magnitudes of IFN-γ production in the case of *Listeria monocytogenes* infection (53). In the case of Mtb infection, the scenario turns out to be more intriguing because TIM3 might function as both an inhibitory molecule of Th1 cell immunity and an enhancer of macrophage activation against the intracellular pathogens (54–58) (refer to discussion in the next section). These seemingly discrepant findings might indeed highlight that TIM3 plays pleiotropic roles in maintaining immune homeostasis by modulating different immune cells in cell-type- and scenario-dependent manners.

## TIM3 Exerts Distinct Functions Upon Infection Depending on the Cell Types and Ligands

As a type I membrane protein, TIM3 is comprised of an immunoglobulin variable domain, an extracellular glycosylated mucin domain, a single transmembrane domain, and a C-terminal cytoplasmic tail with five tyrosines (59, 60). Although the intracellular function of the cytoplasmic tail remains obscure, it is now recognized that Tyr256 and Tyr263 are key binding sites of HLA-B associated transcript 3 (BAT3), a negative regulator of TIM3 activation (61). Upon binding of ligands, the release of BAT3 from phosphorylated Tyr256 and Tyr263 can activate TIM3 signaling by mechanistically inhibiting the mTORC2-AKT pathway in T cells (62). A variety of ligands with different binding sites have been reported in association with TIM3 activation, including galectin 9 (GAL9) (63), carcinoembryonic antigen cell adhesion molecule 1 (CEACAM1) (64), phosphatidylserine (PtdSer) (65), and high mobility group protein B1 (HMGB1) (66). Emerging studies have shown that these molecules can be expressed by different immune cells and bind to different regions on the TIM3 extracellular immunoglobulin V domain, subsequently exerting various biological functions with a cell-type and context-dependent manner. With a focus on the interaction of T cells and invasive pathogens, multiple lines of evidence supports that the binding of TIM3 to GAL9 or CEACAM1 will trigger the release of BAT3 from its intracellular tail and induce Th1 cell inhibition or Treg cell activation, which significantly dampens the INFγ-mediated Th1 response. On the other hand, the bindings to PtdSer and HMGB1, which are regarded as typical markers of apoptosis or cell debris, likely facilitate the engulfment and phagocytosis of apoptotic cells and debris by the TIM3-expressing myeloid cells, like DCs and macrophages (38, 42, 44). Of note, the biology of TIM3-binding to PtdSer might require further in-depth research as it displays much lower affinity compared with other TIM family members (67).

As the first revealed pathway, the TIM3-GAL9 axis negatively regulates Th1-dependent immune responses by suppressing T cells’ production of IFNγ (68) and promoting that of IL10 (49). Consequentially, it dampens the T cell-mediated inflammatory response to pathogens stimulation. However, recent studies have yielded “discrepant” conclusions that TIM3-GAL9 interaction triggers pro-inflammatory response to eliminate pathogens under certain conditions of infection. First, Jayaraman et al. revealed a reciprocal axis of TIM3-GAL9 between macrophages and T cells in a murine model of Mtb infection (54). The binding of T cell’s TIM3 to macrophage’s GAL9 stimulates the macrophage’s bactericidal activity by enhancing caspase-1-dependent secretion of IL1β, independent of the production of IFNγ and inducible NO synthase (iNOS). Unlike its apoptosis-inducible effect on T cells, TIM3-GAL9 interaction does not cause cell death of the affected macrophages, indicating a bidirectional role of TIM3 in regulating the innate and adaptive immune response (54). Indeed, TIM3 expression on the side of the innate cells might oppositely promote the tissue inflammation by enhancing TNFα secretion (33). Consistently, it has been reported in human studies that TIM3-GAL9 interaction activates macrophage-mediated inflammatory response against Mtb infection, which at least partly functions through IL1β secretion (57). It therefore suggests that activation of the TIM3-GAL9 axis can potentially initiate tissue inflammation, at least from the macrophage part.

Based on the recent findings, the TIM3-GAL9 axis seems to play a more complex role in regulating the T cell compartment in response to Mtb infection than macrophage. In animal studies, TIM3^+^PD1^–^ T cells in the early stage of infection are featured by a typical phenotype of effector Th1 cells with enhanced production of IL2, TNFα, and IFNγ. However, TIM3^+^PD1^+^ T cells, which emerge late in chronic infection and resemble a subset of dysfunctional T cells, dampen Mtb clearance by a robust production of inhibitory cytokines, such as IL10. Accordingly, TIM3-targeting intervention is helpful to restore T cell function and improve bacterial control in chronically infected animals (58). These findings reveal the importance of the disease stage in evaluating the role of TIM3 in Mtb infection. In line with the animal studies, elevated TIM3 expression leads to decreased IFNγ production of CD8^+^ T cells in patients with active/severe Mtb infection, which can be restored by TIM3-blocking interventions (55). Intriguingly, early studies have reported that TIM3-expressing T cells are featured by a robust production of IFNγ and TNFα and augment the anti-Mtb activity of macrophages in active TB patients (56). These findings are indeed complementary to our understanding of TIM3 biology that the TIM3-GAL9 axis might exert distinct regulatory functions on T cell and macrophage in two different ways, which are likely associated with the disease stage and microbial activity. Of note, the kinetics of microbial antigen presentation is synergistically determined by the timing of protein translation rather than a simple bacterial load or the number of the TIM3 expressing T cells (69). The functioning pattern of TIM3 might explain the current “inconsistent” data in the Mtb studies that were actually derived from multiple disease models with different phases of Mtb infection. Considering the diversity of TIM3-expressing cell types and their ligands, it is reasonable to posit that TIM3 acts as a double-edged sword in regulating both innate and adaptive immune cells upon pathogen stimulation with a timing-specific manner. We summarize the findings about the role of TIM3 biology in infectious disease with a specific evaluation of its ligands and the expressing cell types (**Table 1**).

**Table 1:** The role of TIM3 in infectious diseases.

Disease model	Cell types	Ligand	Interact with	Infection phase	Subject	Function of TIM3	Ref.
M. Tuberculosis (Mtb)	CD8+T	Galectin-9	Macrophage	Chronic	Murine	triggers IL-1β production by macrophages and limits intracellular Mtb replication. restrains TIM3+ effector T cell responses.	(54)
	CD8+T	Galectin-9	Macrophage	Chronic	Murine	co-express with other inhibitory receptors, marking the subset of effector T cell that is functionally exhausted. TIM3 blockade restores T cell function and improves bacterial control.	(58)
	CD4+T CD8+ T	–	Macrophage	Chronic	Human	limits intracellular Mtb replication TIM3+T-cell subsets express much higher levels of phosphorylated signaling molecules (p38, stat3, stat5, and Erk1/2)	(56)
HIV	CD8+T	–	–	Chronic	Human	TIM3 levels are positively correlated with viral load and disease progression marks the dysfunctional subset of CD8+ T cells in HIV patients TIM3 blockade can restore the proliferative response of TIM3+ CD8+ T cells to HIV-1 peptides *in vitro*	(48)
	CD4+T	Galectin-9	–	–	Human	lowers the expression of the HIV co-receptors CCR5, CXCR4 and α4β7 on the T cells, thus enabling resistance to HIV infection.	(70)
HCV	CD4+T CD8+T	–	–	Chronic	Human	marks dysfunctional T cell population TIM3 blockade can restore the T cell response to HCV infection by enhancing T-cell proliferation and gamma interferon production	(45, 46)
HBV	CD4+T CD8+T	Galectin-9	Kupffer cell	Acute and Chronic	Human	marks the dysfunctional T cell population Tim-3 blockade results in enhanced expansion of HBV-specific CD8 T cells able to produce cytokines and mediate cytotoxicity *in vitro*.	(71)
	CD4+T CD8+T	–	–	Chronic	Human	over-expression of Tim-3 is involved in disease progression of hepatis B and contributes to persistency of infection	(47)
Listeria monocytogenes	Macrophage	–	–	Chronic	Murine	dampens macrophage phagocytosis by inhibiting the Nrf2-CD36/HO-1 signaling pathways. increases bacterial burden/infection tolerance during chronic infection.	(72)
	CD8+T			Acute	Murine	enhances CD8 T cell responses to acute Listeria monocytogenes infection.	(53)
HSV-1	CD8+T	Galectin-9	Neuronal cells	Chronic/latent	Murine	Galectin-9/Tim-3 interaction is responsible for reduced CD8+ T cell effector function.	(73)
LCMV	CD8+T	–	–	Chronic	Murine	Co-expression of Tim-3 and PD-1 is associated with more severe CD8 T-cell exhaustion	(49)

Nrf2, nuclear factor erythroid 2 related factor; HO-1, heme oxygenase-1; HSV-1, herpes simplex virus-1; LCMV, lymphocytic choriomeningitis virus.

## TIM3 Signaling Participates in Acute and Chronic Kidney Inflammation

A growing body of evidence has revealed that TIM3 is a key regulator in kidney immune cells during acute kidney injury (AKI). In macrophages, TIM3 expression activates the TLR-4/NF-κB signaling and exacerbates the kidney inflammatory response to acute ischemia/reperfusion (IR) injury, which can be beneficially ameliorated by an anti-TIM3 strategy (74). Distinctly, TIM3 expression by CD4+CD25+ Treg cells exerts regulatory functions that might be potentially dampened in AKI patients with aggravation of the kidney inflammatory response (75). In the context of kidney transplant, multiple lines of evidence demonstrates the association between aberrant TIM3 expression and acute rejection due to the enhanced inflammatory status in dysfunctional allografts (76–83). These studies consistently support that TIM3, whether expressed in blood, urine, or biopsy samples, can serve as a promising biomarker for monitoring the immune status of the kidney transplant and a potential target of early therapeutic interventions on acute and chronic allograft rejection.

CKD is currently recognized as a type of systemic chronic inflammatory disease (84, 85). Consistent with its role in AKI, TIM3 promotes the inflammatory phenotype of renal macrophages by activation of NF-κB signaling, contributing to podocyte injury in the progression of diabetic kidney disease (DKD) (86). As discussed above, the binding of GAL9 to TIM3 likely exerts pro-inflammatory action on the macrophage side. Supporting this finding, an elevation of serum GAL9 can be detected in patients with severe DKD and positively correlated to their loss of residual renal functions (87). A variety of immune cells, including the T cells and the macrophages, undergo dynamic phenotypic changes and participate in the entire process of inflammation during AKI to CKD progression (3, 88, 89). These overactivated immune cells migrate into the involved kidneys through the circulatory system and participate in progressive inflammatory injury that ultimately leads to loss of renal function. Considering the significant changes among different disease stages, in-depth research with time-course analysis remains necessary to clarify how TIM3 functionally affects different effector cells and their crosstalk at every point of concern in CKD development.

In the setting of autoimmunity, aberrant TIM3 expression is closely correlated with the development of systemic lupus erythematosus (SLE) (90–95), multiple sclerosis (96, 97), and rheumatoid arthritis (98). However, the role of TIM3 underlying different autoimmune diseases has yet to be explored. In the kidney, renal expression of TIM3 can be detected in most lupus nephritis (LN) and positively correlated to the disease activity (99), suggesting the participation of TIM3 in kidney inflammation caused by autoimmunity. Mechanistically, early studies revealed that enhanced TIM3-GAL9 engagement interferes with LN development by potentially inducing Treg cells dysfunction (100). Consistently, deficiency of GAL9 ameliorates disease activity of immune complex glomerulonephritis in a murine model of lupus (101). In addition, data from some other animal models, such as nephrotoxic serum nephritis (102) and anti-glomerular basement membrane glomerulonephritis (103), shed light on the therapeutic benefits of targeting the TIM3-GAL9 axis to treat autoimmune kidney disease. Furthermore, emerging clinical evidence reveals that abnormal TIM3 expression patterns can also be detected in patients with IgA nephropathy (104) or membranous nephropathy (105). Taken together, TIM3 exerts different functions on a variety of immune cells to regulate kidney inflammatory response. Upon binding of GAL9, TIM3 activation probably promotes the inflammatory phenotype of macrophage but dampens the regulatory activity of T cells, thereby leading to aggravation of chronic kidney inflammation. The current findings about TIM-3 biology in kidney diseases are summarized in **Table 2**.

**Table 2:** The role of TIM3 in kidney diseases.

Disease	Cell type of expression	Subject/ model	Function	Ref.
Acute kidney injury	monocytes/macrophages	Murine/ IRI	aggravates kidney IR injury via the TLR-4/NF-κB signaling and NLR-C4 inflammasome activation	(74)
	Treg	Human	lower TIM3 expression in Treg cells restricts the efficacy of Treg response in AKI.	(75)
Kidney transplant	–	Human	Patients of acute and chronic allograft dysfunction have greater urinary and blood TIM3 mRNA expressions. a promising biomarker for noninvasive diagnosis of allograft dysfunction.	(78, 81)
Diabetic kidney disease (DKD)	–	Human; Murine/STZ-induced and db/db mice	TIM3 expression is increased on renal macrophages in DKD aggravates podocyte injury in DKD by promoting macrophage activation via NF-kB/TNF-α pathway.	(86)
	–	Human	Serum GAL9 level in the patient with type 2 diabetes is positively correlated with age, creatinine, urea nitrogen and osmotic pressure and negatively correlated with eGFR.	(87)
IgA nephropathy	–	Human	There is a positive correlation between pathological manifestations and expression degree of TIM3 in IgAN.	(104)
Membranous nephropathy	–	Human/serum	Serum TIM3 concentration in patients with MN is considerably higher than that in healthy individuals. TIM3 may be a diagnostic indicator for distinguishing between healthy individuals and patients with MN as well as between different stages of MN.	(105)
Systemic lupus erythematosus /Lupus nephritis	CD4+T, CD8+T, CD56+T	Human	Expression of TIM3 and Gal9 in serum in patients with SLE are significantly higher than those of healthy controls. The up-regulation of TIM3 and Gal9 expression in patients with SLE is closely related to the SLEDAI scores.	(91)
	CD3+CD4+T, CD3+CD4-T	Human	Expression of Tim-3 and co-expression of TIM3 and Fas on certain peripheral T subsets are associated with disease activity in SLE patients.	(90)
	–	Murine/ pristane-induced model	GAL9 deficiency protects against the development of immune complex glomerulonephritis, arthritis, and peritoneal lipogranuloma formation in pristane-induced lupus model.	(101)
Anti-GBM glomerulonephritis	–	Murine	Administration of GAL9 to anti-GBM GN mice ameliorated renal tubular injury, and reduced the formation of crescents. The protective role of Gal9 in anti-GBM GN is associated with the inhibition of Th1 and Th17 cell-mediated immune responses.	(103)
Myeloperoxidase-ANCA-associated vasculitis	Dendritic cell	Human	Reduced expression of TIM3 and an increased expression of TLR4 are identified on DCs of active MPO-AAV patients. TIM3 plays an important role in maintaining the NETs mediate immune homeostasis in MPO-AAV, suggesting an important role in MPO-AAV development	(106)
Nephrotoxic serum nephritis	Effector Th cell	Murine/NTS	TIM3 is up-regulated in kidneys in NTS and exerts protective roles in the course of disease by suppressing the infiltration of inflammatory cells such as macrophages.	(102)

IRI, ischemiareperfution injury; TLR-4, Toll-like receptor 4; NF-κB, nuclear factorκappa B; NLR-C4, Nod‐like receptor NLR family CARD domain‐containing protein 4; STZ, Streptozotocin; TNF-a, Tumor Necrosis Factor-α; eGFR, estimated glomerular filtration rate; anti-GBM, anti-glomerular basement membrane; MPO-AAV, myeloperoxidase antineutrophil cytoplasmic antibody-associated vasculitis; NETs, neutrophil extracellular traps.

## A Speculative Model of the TIM3-GAL9 Axis in Chronic Kidney Inflammation

**Figure 2:**
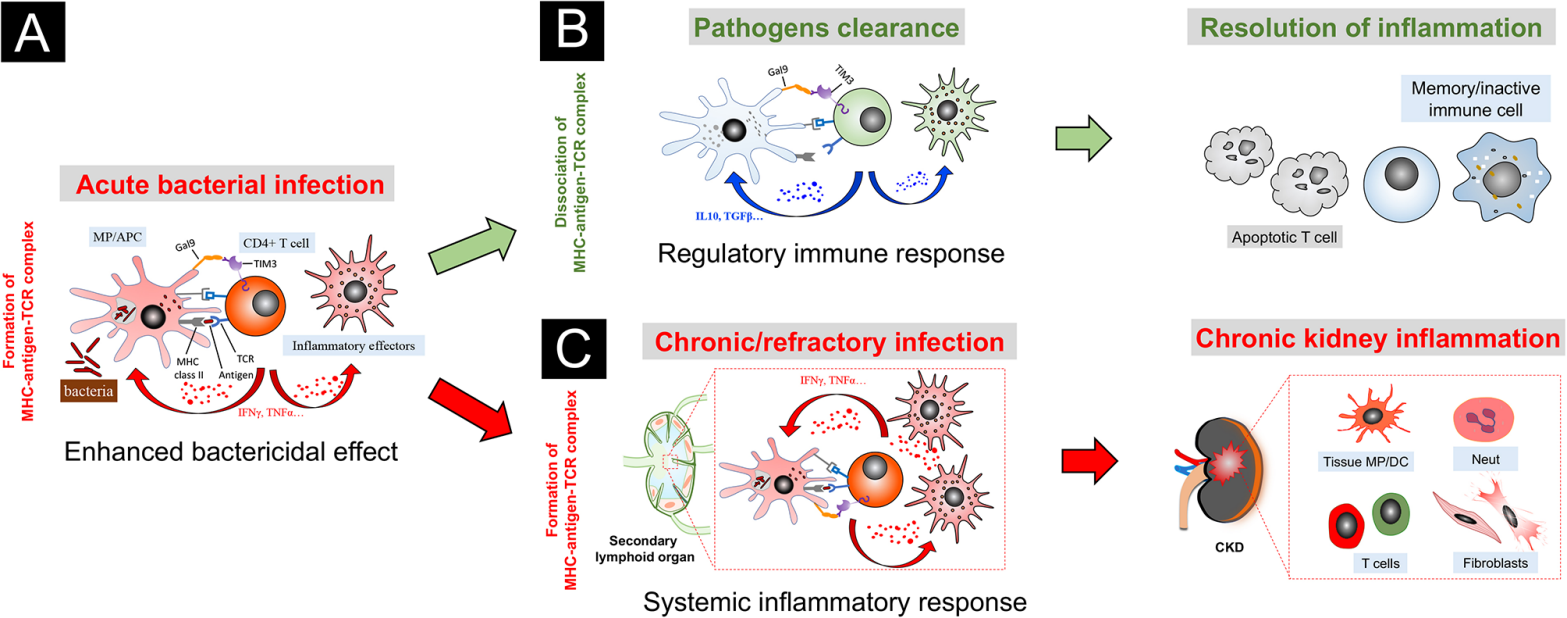
A speculative model of TIM3-GAL9 axis in chronic kidney inflammation triggered by bacterial infection. **(A)** In acute stage of infection, bacterial antigens are presented to T cells by macrophage (MP) or antigen-presenting cell (APC). Upon activation, the primed T cells proliferate with TIM3 induction. With the synergic action of MHC II-antigen-TCR complex, TIM3-GAL9 interaction promotes the effector T cells that secrete abundant pro-inflammatory cytokines, like TNFα and IFNγ, to enhance bactericidal capacities of the phagocytes. Likewise, these paracrine cytokines induce activation of peripheral effector cells and amplify the systemic inflammatory response. **(B)** Upon pathogens clearance, the MHC-antigen-TCR complex unravels. TIM3-GAL9 axis, in turn, exerts an inhibitory action on T cells by induction of regulatory cytokines (like IL10 and TGFβ). In this context, TIM3-GAL9 interaction facilitates the resolution of tissue inflammation by induction of T cells apoptosis and transition of memory immune cells. **(C)** In the case of chronic/refractory infection, the immune system fails to eliminate the intracellular bacteria. The synergy of the MHC-antigen-TCR complex with the TIM3-GAL9 axis persistently stimulates the activation of peripheral immune cells, which are attracted into the involved kidneys of CKD by diseased renal microenvironment and participate in progressive kidney inflammation.

Based on the current findings, we propose a novel model of TIM3-GAL9 interaction in chronic kidney inflammation (**Figure 2**). Upon acute infection, exogenous pathogens are taken up by dendritic cells and macrophages, which traffic to draining lymph nodes and prime T cells *via* the process of antigen presentation. Once activated, the primed T cells proliferate with enhanced TIM3 expression and recruit back to the “crime sense”, where they cooperate with the tissue-resident macrophages *via* the binding of their extracellular TIM3 to the GAL9 on the “involved” phagocytes. With the synergic action of MHC-antigen-TCR complex, TIM3-GAL9 interaction promotes effector T cells that secrete abundant pro-inflammatory cytokines, such as TNFα and IFNγ, to enhance bactericidal capacities of the phagocytes. Likewise, these paracrine cytokines promote activation of peripheral monocyte/macrophage and amplify the systemic inflammatory response. At this moment, the TIM3-GAL9 axis exerts pro-inflammatory functions to enhance the bactericidal capacities of immune cells. In most cases, the active effector cells can eliminate intracellular pathogens, leading to successful infection control. The antigen-TCR complex thereby unravels after pathogens clearance. Without the mutual effect of the microbial antigen complex, the TIM3-GAL9 axis plays an inhibitory role in the TIM3-expressing T cells, which in turn exert regulatory functions on the effector immune cells by their robust secretion of anti-inflammatory cytokines (like IL10 and TGFβ). Meanwhile, the TIM3-GAL9 axis facilitates the resolution of tissue inflammation by induction of T cells apoptosis and the transition of memory immune cells.

We speculate that this TIM3 feedback loop acts through an antigen stimulating- and time length-dependent manner. In the acute phase of infection, the conjunct action of the MHC-antigen-TCR complex is prominent due to bacteria multiplication. It thereby enhances the inflammatory action of the TIM3-GAL9 axis. Once the infection is under control, TIM3-GAL9 interaction switches to exert the regulatory effect on T cells without the synergic action of exogenous antigens. However, in some chronic/refractory infection cases, the immune system fails to restrain the intracellular pathogens, which constitute a source of persistent antigenic stimulation. Instead, it continuously upregulates the TIM3-mediated inflammatory response and undermines the regulation mechanism between the effector T cells and innate immune cells. Due to the excessive inflammatory response, the overactivated effector cells migrate throughout the body *via* the circulation system. It is worth noting that the kidney with abnormal tissue microenvironment in CKD is an organ that the reactive immune cells tend to visit. As a result, the pathogenic activation of TIM3 signaling, triggered by the continuous stimulation of the MHC-antigen-TCR complex, promotes systemic inflammation and ongoing kidney recruitment of multiple pro-inflammatory effectors, including myeloid cells, lymphoid cells, and fibroblasts, which aggravate inflammatory kidney injury and ultimately result in renal fibrosis. This speculative model of TIM3 biology might to some extent explain why chronic/recessive infection is a critical risk factor leading to recurrence or aggravation of CKD (107), especially vigilant for nephrotic syndrome (108) and IgA nephropathy (109, 110).

## Questions and Perspectives

The major function of the immune system in health is to protect the host from infections by initiating a sufficiently strong inflammatory response to pathogens and to avoid excessive immunity against self. The immune system is governed by central and peripheral feedback mechanisms, which provide the necessary inflammatory signals upon encountering microbial antigens and meanwhile trigger feedback suppression during the intensive inflammatory response. TIM3 is widely expressed in a variety of immune cells and capable of binding to different ligands. With its unique structure and biological features, TIM3 potentially possesses some advantages to serve as a multifunctional molecule in the regulation of immune homeostasis. TIM3 can exert substantial actions tuning the inflammatory response to various disease settings by working in synergy with other co-receptors. Emerging evidence has highlighted TIM3 as a stimulatory molecule for optimal immune response rather than a one-way dominant inhibitory receptor. Recent advances on the TIM3-GAL9 axis, which seem to contest the previous findings on its well-established inhibitory roles, actually supplement our understanding of TIM3 biology. The current research has reached consistent conclusions on the TIM3 function in kidney disease that can be cross-referenced with other fields. This review attempts to propose a novel model based on our intriguing clincal findings and current knowledge to reconcile the seeming “discrepancy” of TIM3 biology, particularly the findings related to human infectious disease and CKD progression.

It is important to note that our understanding of TIM3 function in kidney homeostasis is limited. For example, the regulatory mechanism of TIM3 in both innate and adaptive immune cells has yet to be clarified in CKD progression. Furthermore, it remains difficult to exactly speculate as to how TIM3-expressing cells can discriminate the hidden signals presented by various ligand bindings in the kidney. In-depth research is urgently required to address the molecular role of TIM3 in the crosstalk between immune cells and kidney resident cells (like tubular epithelial cells) in the pathogenesis of chronic kidney inflammation, particularly with a focus on the infection-triggering mechanism. Future advances in the understanding of TIM3 biology will help to develop effective strategies in an individualized manner to restore the tissue inflammatory homeostasis in kidney diseases. For nephrologists, it is important to dialectically assess the therapeutic value of the TIM3-targeting strategy in consideration of the timing, the signaling intensity, and the difference in kidney disease settings.

## Data Availability Statement

The original contributions presented in the study are included in the article/supplementary material, further inquiries can be directed to the corresponding author.

## Author Contributions

GC conceived the review and revised the manuscript. HC, CW, and CL together drafted the manuscript. CL, CW, and FY collected clinical information. CL provided statistical and graphic editing support. JL and HL advised on data analyses. All authors read and approved the final manuscript.

## Funding

This work was supported in part by grants from the National Natural Science Foundation of China and Hunan Provincial Natural Science Foundation of China to HC (81970804, 2020JJ4126) and GC (81770691, 82170759).

## Conflict of Interest

The authors declare that the research was conducted in the absence of any commercial or financial relationships that could be construed as a potential conflict of interest.

## Publisher’s Note

All claims expressed in this article are solely those of the authors and do not necessarily represent those of their affiliated organizations, or those of the publisher, the editors and the reviewers. Any product that may be evaluated in this article, or claim that may be made by its manufacturer, is not guaranteed or endorsed by the publisher.
